# Reimagining digital mental health literacy from the Global South: a call for epistemic justice and innovation equity

**DOI:** 10.3389/fpsyt.2025.1611988

**Published:** 2025-08-13

**Authors:** Akhil P. Joseph

**Affiliations:** ^1^ Marian College Kuttikkanam Autonomous, Kuttikkanam, India; ^2^ Christ (Deemed to be University), Bengaluru, India

**Keywords:** digital mental health literacy, epistemic justice, Global South, participatory action research, cultural psychiatry

## Introduction

The integration of digital technologies into mental health systems globally has prompted a momentous reconfiguration of how psychiatric services are conceptualized, accessed, and delivered. From AI-assisted diagnostics and app-based cognitive behavioural therapy modules to digitally mediated peer support platforms and chatbots, these technologies are frequently heralded as scalable innovations capable of addressing longstanding disparities in mental health service provision (1, 2). However, such optimism is often framed within a techno-solutionist paradigm that neglects the structural inequalities that mediate access to digital resources. Without critical scrutiny of how digital infrastructures intersect with sociocultural and political conditions, the integration of digital tools into psychiatric care risks deepening rather than redressing global health disparities (3). The digital divide—manifested not only in differential access to technology but also in disparities in digital fluency, literacy, and cultural—poses significant barriers to equitable care (4). This disparity is compounded by a lack of engagement with context-specific epistemologies that shape how mental health is experienced, narrated, and addressed in diverse sociocultural contexts, particularly across the Global South. The present manuscript outlines a research protocol for a conceptual and empirical reframing of digital mental health literacy (DMHL) using a participatory and culturally grounded methodology, with particular focus on research design, implementation strategies, and ethical considerations.

## Rethinking digital mental health literacy in the Global South

Digital mental health literacy (DMHL) must be reconceptualized as a context-specific and relational construct that reflects the sociocultural, linguistic, and relevance spiritual dimensions shaping mental health engagement in diverse communities across the Global South (5). While conventionally defined by Western biomedical norms as the individual ability to meaningfully engage with digital tools and access mental health content in dominant languages, this framework erases alternative epistemologies and delegitimizes embodied, symbolic, and communal pathways to care (6). Culturally grounded paradigms—such as the Pacific model emphasizing the triadic relationship between Atua (God), Tagata (people), and laufanua (land); the Yoruba worldview rooted in ancestral alignment; or Quechua healing traditions that blend storytelling and nature immersion—demonstrate that mental health is fundamentally relational, place-bound, and spiritually infused (7–9). These ontologies challenge technocentric models that reduce DMHL to usability metrics or clinical literacy. The dominant reliance on standardized digital mental health frameworks privileges Eurocentric norms, obscures epistemic diversity, and enforces conformity under the guise of universality (10). This epistemological reductionism not only distorts the validity of community-based knowledge but has material consequences in shaping which interventions receive funding, which populations are deemed “reachable,” and which modalities of healing are institutionalized. If digital psychiatry is to evolve into an inclusive and contextually grounded discipline, its universalist assumptions must be dismantled and replaced with pluralistic, equity-driven frameworks that recognize multiple ways of knowing, communicating, and healing (11).

## Community-based innovations and epistemic alternatives

In contrast to dominant paradigms that universalize DMHL through Eurocentric frameworks, a robust and expanding body of empirical evidence from the Global South underscores the emergence of grassroots digital mental health innovations that are deeply grounded in local sociocultural realities, epistemologically diverse in nature, and structurally embedded within existing community dynamics (12). These innovations are not peripheral or reactive adaptations to externally imposed models but rather exemplify novel and autonomous digital paradigms that originate from and are co-produced by communities themselves, directly challenging the epistemic authority of standardized digital psychiatry (13). In India, WhatsApp-based psychoeducational campaigns conducted in regional languages draw upon culturally specific idioms of distress and local wellness concepts to foster communal learning and peer support (14, 15). In sub-Saharan Africa, the development of mobile applications incorporating audio-visual storytelling effectively circumvents literacy and language barriers while validating indigenous knowledge systems and affective communication modes (16). In Brazil, Afro- Brazilian youth collectives are leveraging digital platforms not only to resist dominant biomedical framings of distress but to articulate mental health through lenses of racial justice, historical trauma, and political resistance (17). These models represent more than just context-specific interventions—they constitute coherent, scalable, and empirically substantiated epistemological alternatives that redefine the very contours of what DMHL can and should encompass when informed by equity, inclusion, and cultural legitimacy (18).

## Challenging dominant notions of scalability

The dominant framing of scalability in global mental health must be fundamentally redefined to reject the flawed premise that uniformity equates to effectiveness, and instead affirm that meaningful scalability requires the capacity to adapt to diverse cultural, epistemological, and infrastructural contexts without compromising integrity or local relevance. Hyperlocal and community-embedded models are successful precisely because they reflect and respond to the complex interplay of symbolic systems, sociopolitical histories, and relational dynamics that shape mental health experiences in their respective settings. These models do not diminish clinical validity; rather, they substantively enhance therapeutic efficacy by fostering culturally situated trust, facilitating experiential resonance, and enabling user engagement that is both ethically grounded and emotionally meaningful. For example, a mobile-based radio drama series developed in rural Uganda that incorporates local idioms and culturally grounded narratives of mental illness has proven more effective in increasing mental health literacy and help-seeking behaviors than imported psychoeducational video content delivered in English. Their impact is not speculative—empirical studies consistently demonstrate that such initiatives lead to increased help-seeking behaviors, reductions in stigma, improved treatment adherence, and elevated mental health literacy among populations that are typically underserved or rendered invisible in mainstream psychiatric paradigms. The failure of global mental health policies to formally recognize, fund, and institutionalize these contributions represents an entrenched form of epistemic injustice that not only undermines the potential for inclusive innovation but also reinforces the structural marginalization of knowledge systems that are vital to transforming mental health outcomes on a global scale.

## Research priorities and epistemic shifts

To address these challenges, a paradigmatic shift in how we conceptualize, operationalize, and evaluate DMHL is essential—not as a matter of theoretical refinement, but as a necessary corrective to the epistemic and structural limitations embedded within existing models. This shift must be guided by a research agenda driven by three interlinked and empirically actionable questions: First, how do community-embedded digital mental health practices across the Global South redefine the conceptual boundaries and functional applications of DMHL in ways that are both culturally specific and clinically effective? Second, what forms of digital engagement and knowledge transmission emerge from marginalized contexts, and how do these practices illuminate the limits of conventional literacy-based paradigms in global psychiatry? Third, how can the co-production of DMHL tools and interventions by historically excluded populations contribute to equitable, scalable, and context-sensitive models of mental health care that are both epistemologically plural and systemically integrative? To distinguish this framework from existing co-production paradigms, this approach underscores a shift from participatory inclusion to epistemic re-centering, foregrounding community-originated systems of meaning. This approach emphasizes not only the participatory dimension but also the radical epistemic repositioning it requires -centering not just end-user feedback but locally rooted systems of knowledge production as foundational to the design, governance, and evaluation of digital tools (19). Addressing these questions requires integrating participatory action research (PAR) as a core methodology, alongside ethnographic action research, critical case studies, and system dynamics modeling, to examine how cultural, infrastructural, and political factors shape digital mental health engagement. PAR is especially suited to this task because it centers community agency and experiential knowledge while enabling the identification of vernacular digital pedagogies—locally grounded and culturally resonant modes of knowledge sharing such as storytelling, intergenerational dialogue, and symbolic or visual expression. Rooted in emancipatory and praxis-oriented traditions, PAR involves iterative cycles of collective inquiry, reflection, and action that prioritize the co-generation of knowledge with, rather than about, marginalized communities. These insights, when embedded into intervention design, produce emotionally and linguistically meaningful tools that challenge the dominance of institutionalized formats (20). As a method grounded in epistemic justice, PAR not only diversifies what counts as valid evidence but also contributes to building socially responsive and scientifically robust digital psychiatry frameworks attuned to marginalized realities.

## Phased implementation and ethical evaluation frameworks

To institutionalize the gains from PAR-informed models, a phased implementation strategy is required, structured around iterative cycles of community engagement, co-design, policy integration, and evaluative refinement. In Phase I, ethnographic mapping and community consultations would document existing grassroots DMHL practices, attending to the cultural semiotics, symbolic ecologies (the culturally embedded systems of meaning, narratives, and metaphors that shape how communities understand and respond to mental health challenges), and sociohistorical determinants that inform local digital literacies. Phase II would involve the participatory co-development of digital tools and interventions, ensuring that these are not only technologically functional but also ethically resonant and epistemologically congruent. Phase III would focus on embedding these tools within existing mental health infrastructure, supported by multisectoral partnerships, regulatory alignment, and capacity-building initiatives. Throughout, evaluative frameworks must be expanded beyond traditional clinical endpoints to include qualitative and mixed-methods indicators such as narrative coherence, intersubjective trust, and symbolic alignment.

Evaluation within a reconceptualized digital mental health framework must decisively move beyond exclusive reliance on standardized instruments like the PHQ-9 and GAD-7, which— despite their clinical utility—remain inadequate for capturing the symbolic, affective, and culturally mediated dimensions of psychological experience across diverse ecologies. Instead, assessment practices must be recalibrated to center community-relevant outcomes such as stigma reduction, transformation of collective narratives, epistemic self-recognition, and the creation of culturally legitimate pathways to care. However, such a pluralistic approach also raises critical ethical concerns, including the risk of cultural appropriation, tokenism, and the instrumentalization of local knowledge systems without long-term accountability or structural redistribution. To mitigate these risks, evaluative paradigms must not only integrate participatory and narrative methodologies, but also be grounded in sustained, ethically governed partnerships that prioritize relational accountability and consent. Institutionalizing this shift demands more than methodological expansion; it necessitates a recalibration of epistemological commitments and governance mechanisms to ensure that community-authored frameworks are not co-opted by dominant institutions. Only through such ethically grounded epistemic pluralism can digital psychiatry become not merely inclusive in theory but equitably transformative in practice.

The future of digital mental health depends on a globally coordinated research and implementation agenda that reconfigures its epistemic foundations, not merely extends technological efficiency. This transformation requires sustained commitments to epistemic humility, cognitive plurality, and distributive justice across disciplines, institutions, and geopolitical contexts. Institutions in the Global North must move beyond transactional funding to embrace reciprocal knowledge-sharing, shared governance, and equitable partnerships with communities in the Global South. Future research must prioritize repositioning communities in the Global South not as passive recipients but as epistemic co-authors capable of generating digital mental health frameworks that emerge from their own cultural logics and ontological traditions. This necessitates inquiry into how such frameworks can be institutionally recognized, integrated, and scaled without epistemic distortion or extractive appropriation. There is also a critical need to investigate how current academic infrastructures—such as journal editorial policies and conference programming—can be restructured to support narrative inquiry, decolonial methodologies, and pluriversal knowledge systems. Research must also explore governance mechanisms that mitigate gatekeeping practices and enable ethical co-authorship across geopolitical divides. This agenda calls for interdisciplinary investigations into the conditions necessary for epistemic redistribution, equitable knowledge partnerships, and structural accountability in digital psychiatry. Only through such a sustained and critically reflective research agenda can inclusive, contextually grounded, and ethically coherent DMHL frameworks be developed, validated, and institutionalized on a global scale.
